# Sublethal Effects of Cadmium on the Osmoregulatory and Acid-Base Parameters of Tilapia (*Oreochromis niloticus*) at Various Times

**DOI:** 10.1155/2023/2857650

**Published:** 2023-02-27

**Authors:** Agoes Soegianto, Bambang Yulianto, Carolyn Melissa Payus, Moch Affandi, Wildanun Mukholladun, Khudrotul Nisa Indriyasari, Ary Marchellina, Nailul Muthiati Rahmatin

**Affiliations:** ^1^Department of Biology, Faculty of Science and Technology, Universitas Airlangga, Surabaya, Indonesia; ^2^Department of Marine Science, Faculty of Fisheries and Marine Science, Diponegoro University, Semarang, Indonesia; ^3^Faculty of Science and Natural Resources, Universiti Malaysia Sabah, Kota Kinabalu, Sabah, Malaysia

## Abstract

**Background:**

Cadmium (Cd) can contaminate aquatic environments as a result of anthropogenic activity. Cd accumulates quickly in the tissues of fish and has the potential to affect their physiology, including osmoregulation and acid-base balance. Therefore, the purpose of this study was to examine the sublethal effects of Cd on the osmoregulation and acid-base balance of tilapia *Oreochromis niloticus* at different times.

**Methods:**

Fish were exposed to sublethal concentrations of Cd (1 and 2 mg/L) for 4 and 15 days. At the end of the experiment, fish were collected from each treatment to examine the levels of Cd and carbonic anhydrase (CA) in the gills, plasma osmolality, ions, blood pH, pCO_2_, pO_2_, and hematological parameters.

**Results:**

Cd concentrations in gills rose with increasing Cd concentrations in the medium and exposure time. Cd inhibited respiration by generating metabolic acidosis, decreasing gill CA, reducing pO_2_, plasma osmolality, Cl^−^, and K^+^, particularly at 2 mg/L for 4 days and 1 and 2 mg/L for 15 days. Red blood cell (RBC), hemoglobin (Hb), and hematocrit (Ht) levels decreased as Cd levels in water and exposure duration increased.

**Conclusion:**

Cd inhibits respiration, lowers RCB, Hb, and Ht levels and decreases ionic and osmotic regulation. All of these impairments can limit a fish's ability to provide appropriate oxygen to its cells, hence diminishing its physical activity and productivity.

## 1. Introduction

Cd is a hazardous heavy metal that may accumulate in organisms and the environment for extended periods of time. Cd is often found in aquatic systems due to both natural and human-caused activities [1]. Natural waters commonly have less than 1 *μ*g/L of Cd [2]. It may reach 2 mg/L in contaminated water [3]. Cd accumulates rapidly in the organs and tissues of rainbow trout *Onchorhynshus mykiss* [4] and tilapia *Oreochromis niloticus* [5] and may diminish growth; affect behavior, plasma ion balance, and respiration of *O. mykiss* [6], *O. niloticus* [7], and carp *Cyprinus carpio* [8]; suppress hormone activity of Prussian carp *Carassius auratus gibelio* [9] and Neotropical fish *Rhamdia quelen* [10]; inhibit reproduction of red tilapia *Oreochromis* sp [11] and neotropical fish *Prochilodus magdalenae* [12]; lower hematological, biochemical parameters; and disrupt enzyme function of *O. niloticus* [13, 14].

As mentioned above, Cd inhibits a variety of biochemical and physiological functions in fish. However, relatively few studies have been conducted on how Cd affects fish acid-base balance. Prior research has demonstrated that certain metals (such as copper, lead, and zinc) disrupt the acid-base balance of a number of fish species such as rainbow trout *Salmo gairdneri* [15], *O. mykiss*, [16], groovy mullet *Liza dumerili* [17], and spiny dogfish *Squalus acanthias* [18]. Metals and other pollutants may cause hypoxia and acid-base imbalance by destroying the gills of European ell *Anguilla Anguilla* [19], Atlantic salmon *Salmo salar* [20], and *C. carpio* [21].

Acid-base regulation in fish is linked to ionic regulation because it is mostly based on the constant exchange of H^+^ and HCO_3_^−^ through the gills for Na^+^ and Cl^−^. Carbonic anhydrase (CA) controls the release of CO_2_ and regulates ions and the acid-base balance [20, 22, 23]. *In vitro* studies on fish show that metals inhibit CA of horse mackerel *Trachurus trachurus* [24] and bighead carp *Hypophthalmichthys nobilis* [25]. However, there is a lack of *in vivo* research on the effects of metals on fish CA. Therefore, the effects of Cd on CA in fish will be investigated in this study.

Our study will use *O. niloticus* as a test animal. This fish is a commercially significant species, notably in Indonesia, and it typically responds rapidly to environmental changes [26]. Due to the fact that tilapia is often reared in freshwater that is continually polluted by metals from human activities, the effects of Cd on tilapia are of great concern. In this study, we evaluated the effects of sublethal Cd exposure on acid-base, osmoregulatory, blood parameters, and gill CA in *O. niloticus* over 4 and 15 days.

## 2. Materials and Methods

### 2.1. Protocol for Sample Collection and Laboratory Acclimation

This research used tilapia *O. niloticus* from a fish farm in Pasuruan, East Java that measured 10.1 ± 0.7 cm in length and weighed 16.1 ± 0.5 g. A plastic bag containing oxygenated fresh water was used to bring them to the laboratory. The animals were then acclimated for two weeks in laboratory acclimation tanks (250 L) using dechlorinated tap water at 28-29°C and 12-hour light/12-hour dark photoperiods [26]. A biofilter made up of gravel, sand, and sponge filters, maintained water quality by recirculating the water continuously. Pellet fish meal equal to 1% of the fishes' daily estimated body weight was given to them [26]. To preserve the water quality at an adequate level for fish, excrement, uneaten food scraps, and other undesired things were removed daily. During acclimation and testing, daily measurements showed that the optimal ranges for temperature (28.5 ± 0.6°C), pH (7.9 ± 0.2), and dissolved oxygen (7.4 ± 0.3 mg/L).

### 2.2. Experiment Solution Preparation

A Cd stock solution of 1000 mg/L was prepared by dissolving 2.744 g of Cd(NO_3_)_2_.4H_2_O (Merck, Darmstadt, Germany) in 1 L of deionized water. Our previous study demonstrated that the lethal concentration (96 h LC_50_) and 95% confidence intervals of Cd to *O. niloticus* were 7.53 (6.11–9.28) mg/L [27]. Based on this LC_50_ value the concentrations of Cd used in this experiment were: 1 mg/L (corresponding to 0.86 mg/L measured level), 2 mg/L (equivalent to 1.78 mg/L measured level), and control (equivalent to 0.001 mg/L measured level, does not contain Cd). From an ecotoxicological perspective, the Cd concentrations utilized in this examination might be found in the natural habitat of fish [3]. Our experiments were conducted across two distinct time periods: 4 days (short term) and 15 days (long term).

### 2.3. Investigation of the Effects of Cadmium on Fish

Following the acclimation period 50 physically active fish were randomly picked from the acclimatized holding tank and put into 10 separate tanks, each containing 5 fish. Each tank had a capacity of 40 liters and was filled with testing medium: 1 mg/L of Cd for 4 days and 15 days, 2 mg/L of Cd for 4 days and 15 days, and the control. For each concentration, two tanks were used. Half of the test medium was replaced every 48 hours to keep the Cd content constant. During the experiment, the fish were fed pellet fish meal equivalent to 1% of their estimated daily body weight [26]. At the end of experiment, 5 randomly selected fish were taken from each treatment to collect blood and gill tissue samples. Cd-containing experimental water was collected and kept in a metal waste water storage tank when the experiment was completed. Any procedures that required the use of animals were carried out in accordance with Airlangga University's Animal Care and Use Policy.

### 2.4. Cd Measurement in Gills

Soegianto et al. [28] technique was used to measure Cd in tilapia gills. To attain a uniform weight, tilapia gills were excised and dried in the oven at 65°C for 48 h. Next, the dried gills were pulverized. Following this, 0.5 g of pulverized gills were digested for 4 h at 90°C in 3 mL of H_2_NO_3_ (Merck, Darmstadt, Germany). After the digested gill sample was cooled, it was filtered using Whatman filter paper with a pore size of 0.45 *μ*m and the volume was brought up to 50 mL with deionized water. Concentrations of Cd were determined using a Shimadzu AA-7000 atomic absorption spectrophotometer (Tokyo, Japan), with values expressed in mg/kg dry weight and a limit of detection of 0.001 mg/kg. The analytical method was confirmed by measuring heavy metals in standard reference material (DORM-4) from the National Research Council of Canada. Validation of the analytical method found the Cd recovery to be 106% of the certificate of DORM-4.

### 2.5. Evaluation of Blood Chemistry and Physiological Variables

Before blood was drawn from the fish, a 200 mg/L clove solution was used to sedate them [29]. With a 1 mm plastic syringe, blood was swiftly collected for every fish through the caudal aorta [17]. The blood was then deposited in vacutainer tubes with tripotassium-ethylene diaminetetraacetic acid (EDTA), an anticoagulant. A SFRI Blood Cell Counter 33 (Jean d'Illac, France) was used to measure red blood cell (RBC) numbers, hematocrit (Ht), and hemoglobin (Hb) concentrations [26]. Blood pH, pCO_2_, and pO_2_ were determined using a GASTAT-Navi analyzer (Japan). pCO_2_ and pO_2_ were both given in mmHg. Blood plasma was obtained from blood sample after 10 minutes of centrifugation at 5000 rpm and 4°C. An osmometer (Fiske® 210, Norwood, MA, USA) was used to test the osmolality of 20 *μ*L of blood plasma. The result was given in mOsm/kg. To measure the concentrations of plasma electrolytes (Cl^−^, Na^+^ and K^+^) were carried out using a SpotChem EL SE-1520 (Kyoto, Japan). The results were given in mmol/L [26].

CA performs an essential function in the regulation of acid-base balance in fish, which primarily happens through the gills [30]. Hence, CA level in gills exposed to cadmium were measured in this study. CA levels were measured using enzyme-linked immunosorbent assay (ELISA) kit (Catalog Number E0123Fi) following the manufacturer's instructions (Bioassay Technology Laboratory Biotech Co. Ltd.).

### 2.6. Analyses of Statistical Data

All data was provided as mean and standard deviation and tested for normality. The results were then analyzed using two-way ANOVA and Tukey's HSD. When *p* < 0.05, the statistically significant difference existed. All statistical analyses used IBM® SPSS® Statistics version 25.

## 3. Results

**Figure 1:**
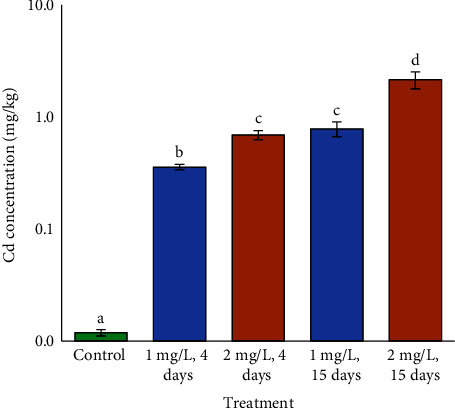
Cd concentration in fish gills exposed to various Cd concentrations in medium. Lower case letters represent significant differences (*p* < 0.05, *a* < *b* < *c* < *d*).

None animals perished during testing. Two-way ANOVA demonstrated that Cd, time, and the interaction of Cd and time had significant effects on Cd accumulation in fish gills (Table 1). Cd in fish gills subjected to Cd is proportional to exposure duration and medium Cd concentration (Figure 1). Fish treated to higher Cd (2 mg/L) would have the greatest concentrations. Gill Cd concentrations were nearly equivalent after 1 mg/L for 15 days and 2 mg/L for 4 days.

**Figure 2:**
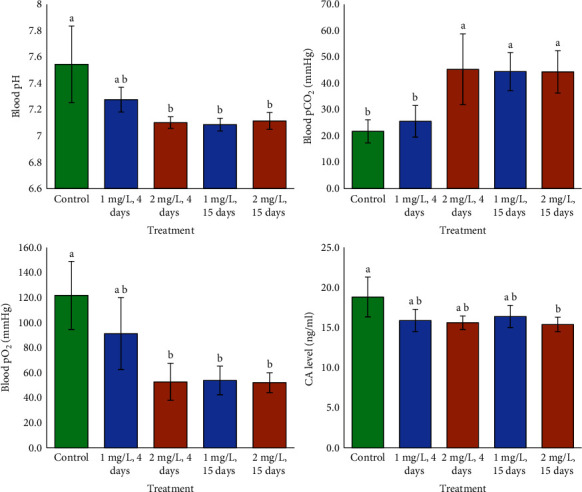
Blood pH, pCO_2_, pO_2_, and CA levels in fish subjected to varied concentrations of Cd in the medium. Lower case letters indicate significant differences (*p* < 0.05, *a* < *b* < *c*).

Cd showed significant impact on pH, pO_2_, and CA; however, neither time nor the combination between Cd and time had significant effects on pH, pO_2_, or CA (Table 1). This experiment demonstrated the significant effects of Cd, time, and their interaction on pCO_2_ (Table 1). Fish exposed to lower Cd (1 mg/L) for 4 days had similar pH and pO_2_ levels as controls. Fish treated to lower Cd for 15 days and higher Cd for 4 days and 15 days showed decreased pH and pO_2_ levels. Blood pCO_2_ was greatest in following exposure to lower Cd for 15 days and higher Cd for 4 and 15 days. Comparing all Cd treatments to the control, Gill CA levels decreased (Figure 2).

**Figure 3:**
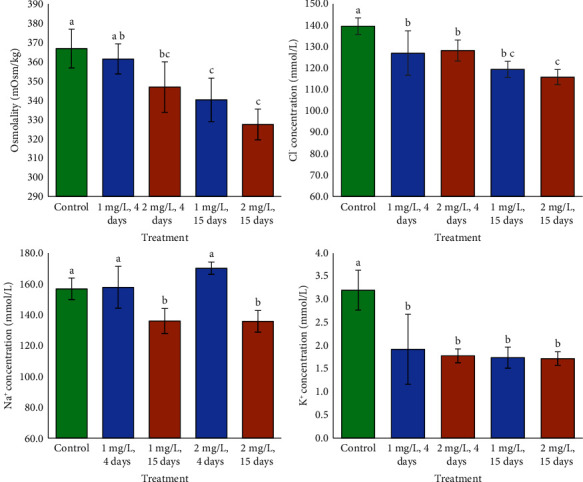
Osmolality and ion levels in fish subjected to Cd exposure. Lower case letters exhibit significant differences (*p* < 0.05, *a* < *b* < *c* < *d*).

The two-way ANOVA revealed that Cd and time had significant effects on plasma osmolality and Cl^−^ levels in tilapia, but there were no significant Cd-time interaction effects (Table 1). Cd, time, and the combination of Cd and time all had a significant effect on plasma Na^+^ levels in tilapia. Cd had a significant effect on plasma K^+^ concentrations, while time and the Cd-time interaction had no effect (Table 1). The plasma osmolality of control fish did not change from those exposed to lower Cd for 4 days, although it was higher than those treated to higher Cd. Reduced plasma osmolality occurred in fish subjected to all Cd concentrations for 15 days. Fish subjected to lower Cd for 4 days and 15 days and higher Cd for 4 days did not substantially alter Cl^−^ levels. Fish treated to higher Cd for 15 days got the lowest Cl^−^. Four days after Cd exposure, fish plasma Na^+^ levels were not different from controls. After 15 days, the plasma Na^+^ content across all Cd-treated fish was considerably lower compared to the control group. Plasma K^+^ contents of fish subjected to all doses of Cd were not substantially different, but lower than in the control group (Figure 3).

**Figure 4:**
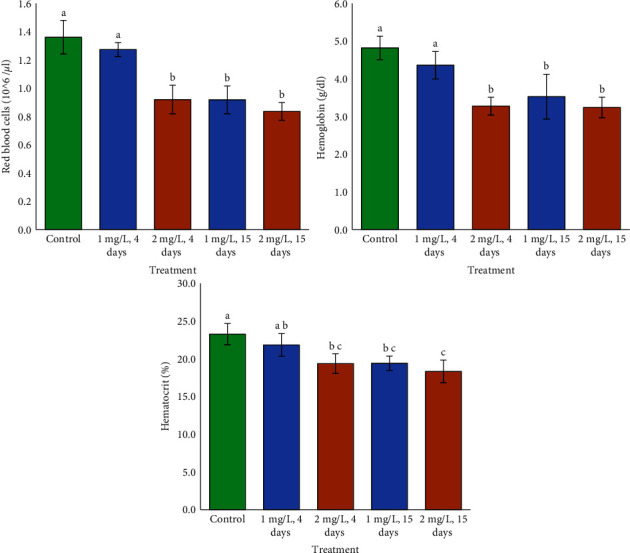
Red blood cells, hemoglobin, and hematocrit in Cd-treated fish. Lower case letters show significant differences (*p* < 0.05, *a* < *b* < *c*).

We reported significant effects of Cd, time, and the combination of Cd and time on RBC and Hb (Table 1). There were no significant interaction effects between Cd and time on Ht; however, both Cd level and time had a significant effect on fish Ht (Table 1). Fish treated to higher Cd for 4 and 15 days exhibited lower RBC, Hb, and Ht levels than that of the control, whereas 1 mg/L for 4 days showed no impact. Fish subjected to lower Cd for 4 days, higher Cd for 4 days, and 15 days had relatively similar RBC, Hb, and Ht levels (Figure 4).

## 4. Discussion

This research made use of a relatively high concentration of Cd since *O. niloticus* has a significant tolerance to Cd. In this investigation, sub-lethal dosages of 1 and 2 mg/L of Cd were used. These concentrations may be higher than those present in the natural environment, but they have been found in contaminated waterbodies [3]. It is expected that examinations into the effects of Cd on the parameters of the blood will be able to be carried out at this concentration, and that the effects will be able to be clearly detected. Variable exposure times and Cd concentrations resulted in varying gill Cd levels in this study's tilapia; 15 days of exposure to a higher Cd concentration was greater than 4 days of exposure to a lower Cd concentration. Severe acidosis occurred due to the higher Cd content and longer exposure period. This ultimately results in an increase in pCO_2_ followed by a decrease in pO_2_, most likely due to gill epithelial disruption as postulated by Spry & Wood [15]. The gill plays a vital role in fish respiratory gas transfer as the major site of CO_2_ sensing and the known site of O_2_ chemoreception [30–32], and this function may be disturbed in Cd-exposed fish. According to the results of our study, Cd exposure caused a decreased concentration of CA in the gills of tilapia. It is believed that this decline is the result of gill cells losing their ability to convert CO_2_ into HCO_3_^−^, which was also found by Larsen et al. [16].

Heavy metals alter monovalent ion regulation, leading ion outflow in freshwater fish and absorption in marine fish [6, 33, 34]. After 4 days exposure to Cd, fish plasma Cl^−^ dropped but Na^+^ remained stable. At this treatment concentration, the acid-base regulation that occurs during hypercarbia may be governed by the interchange of Cl^−^ and HCO_3_^−^ in the gills, whereas the exchange of Na^+^ and H^+^ may play only a small role. Freshwater and marine fishes rely more on Cl^−^-mediated activities more than Na^+^-mediated activities during hypercarbia [35, 36]. This study demonstrated that after 15 days of Cd exposure, ionic-osmotic regulation sharply diminishes. Without a doubt, when prolonged exposure is carried out, it will result in mortality; nevertheless, this will require further investigation.

After 4 days in lower and higher Cd, fish maintained a limited Na^+^ flux, resulting in a decline in only Cl^−^. Similar findings have shown that in environmental hypercapnia, the brown bullhead (*Ictalurus nebulosus*) showed the typically low and transient Na^+^ fluxes, whereas Cl^−^ fluxes are greater and more persistent, resulting in a sharp decrease in Cl^−^ [37]. Atlantic salmon (*Salmo salar*) compensate for a respiratory acidosis associated with 96 h of exposure to hypercapnia in freshwater by increasing strong ion difference, predominantly through a reduction in plasma Cl^−^, presumably via branchial Cl^−^/HCO_3_^−^ exchange [36]. However, after 15 days, the capacity of the tilapia to maintain a low Na^+^ flux was overcome, which resulted in a decrease in the blood levels of both Na^+^ and Cl^−^. Larsen et al. [16] suggested that another possible explanation for the decrease in Cl^−^ is that heavy metal exposure may modify the Na^+^/K^+^-ATPase activity in chloride cells, thereby disrupting the branchial Cl^−^/HCO_3_^−^ exchanger. Copper exposure decreased the osmolality and ion content of hemolymph of shore crab (*Carcinus maenas*) [38]. Similarly, Zheng et al. [39]; demonstrated that polychlorinated biphenyls change the osmoregulatory response of *O. niloticus*. All of these could be a result of alterations in osmoregulating Na^+^/K^+^-ATPase activity [38, 39].

All Cd-treated tilapia exhibited lower K^+^ levels than controls. The lower level of K^+^ in Cd-treated tilapia may be owing to damaged gill epithelium and Na^+^/K^+^-ATPase activity, causing changes passive fluxes. Since fish gills can let K^+^ through, efflux is greater than influx. A decrease in K^+^ uptake, as proposed by Patridge & Lymbery [40]; is more significant than an increase in K^+^ loss. Nussey et al. [41] meanwhile suggested that osmotic adaptation was responsible for the decrease in serum K^+^. The considerable alterations in Na^+^/K^+^-ATPase activity may explain why the blue mussel (*Mytilus edulis*) is unable to compete against the enhanced passive ion efflux that occurs when it is exposed to the antifouling chemical chlorothalonil [42].

Some hematological changes were also associated to acidosis. Kurbel [43] suggested that low plasma pH may have caused Cl^−^ to move into red blood cells, or Cl^−^ may have moved into the intracellular layer to restrict lactate efflux. In addition, Turner et al. [44] observed an increase in blood lactate and a decrease in plasma Cl^−^ in highly trained trout, and they speculated that these changes were due to an exchange. The decline in Ht correlated with erythrocyte shrinkage. In addition to this, there was a correlation between the drop in Ht and the shrinking of the erythrocytes. As shown by tinier carp (*Cyprinus carpio*) red cells, a considerable reduction in blood O_2_-affinity may have resulted from a reduction in the size of large red cells in fish exposed to higher Cd levels and for longer durations. This was observed because larger red cells are more oxygen-binding [45]. There is a potential that the O_2_ transport in the blood would become impeded.

Higher Cd and longer exposure caused in decreases in all blood parameters tested in this study. A disruption in the erythrocytes or erythropoietic function is indicated [46]. Diverse fish species that were exposed to varying concentrations of heavy metals exhibited lowered red blood cells, hemoglobin, and hematocrit levels [26, 47–49].

A significant decrease in RBC amount showed that Cd might destroy RBC during erythrocyte circulation. Heath [50] reportedly discovered something similar. Al-Rudainy [51] found that heavy metals impede the enzymatic pathway that produces Hb in fish. The reductions in RBC, Hb, and Ht show that tilapia experienced anemia or hemodilution. This is consistent with our study's pO_2_ testing, that shows that Cd-treated fish exhibit a considerable drop in pO_2_. Wepener et al. [52] and Nussey et al. [41] proves that when fish are in this state, they can't get enough oxygen to their tissues, which makes them less active and productive.

## 5. Conclusion and Implication

Cd can enter and contaminate aquatic ecosystems as a result of human activities, and once there, it can be stored and accumulated in the aquatic ecosystem or directly absorbed by aquatic organisms. Tilapia *O. niloticus* is a commercially valuable species that provides protein. Due to the fact that tilapia is frequently farmed in freshwater that is continuously contaminated by metals resulting from human activities, the effects of Cd on tilapia require considerable attention. As our study demonstrated that Cd would have an effect on the reduction of ionic and osmotic regulation, acid-base balance, blood O_2_-affinity, blood O_2_ delivery, and the fish's ability to provide adequate oxygen to cells, treating source waters that may be contaminated with Cd before they enter fish ponds or tanks is a practical recommendation that could be implemented during fish cultivation. In addition, by decreasing the accumulation of Cd in farmed fish, we may prevent the negative effects of Cd, such as interference with children's central nervous systems, oxidative stress, cellular death, inflammation, and human renal failure [53].

## Figures and Tables

**Table 1:** Two-way ANOVA of Cd level in gills, osmoregulatory, acid-base, and hematological parameters of *O. niloticus* after to Cd (1 and 2 mg/L) for 4 and 15 days.

Dependent variables	Source	*F*	*p*
Cd in gills	Cd	190.328	≤0.000
	Time	112.041	≤0.000
	Cd-time	53.243	≤0.000

pH	Cd	17.481	≤0.000
	Time	0.843	0.368
	Cd-time	1.029	0.373

pCO_2_	Cd	21.547	≤0.000
	Time	4.289	0.049
	Cd-time	5.016	0.015

pO_2_	Cd	28.092	≤0.000
	Time	2.660	0.116
	Cd-time	2.532	0.101

CA	Cd	10.406	0.001
	Time	0.025	0.875
	Cd-time	0.123	0.885

Osmolality	Cd	21.182	≤0.000
	Time	13.168	0.001
	Cd-time	3.311	0.054

Cl^−^	Cd	30.810	≤0.000
	Time	10.621	0.003
	Cd-time	3.114	0.063

Na^+^	Cd	3.641	0.042
	Time	38.424	≤0.000
	Cd-time	11.055	≤0.000

K^+^	Cd	38.372	≤0.000
	Time	0.273	0.606
	Cd-time	0.122	0.886

RBC	Cd	64.648	≤0.000
	Time	17.812	≤0.000
	Cd-time	9.558	0.001

Hb	Cd	45.597	≤0.000
	Time	4.670	0.041
	Cd-time	4.113	0.029

Ht	Cd	26.741	≤0.000
	Time	5.307	0.030
	Cd-time	1.986	0.159

## Data Availability

The data used to support the findings of this research are available upon reasonable request from the corresponding author.
